# Neuropsychology and MRI correlates of neurodegeneration in SPG11 hereditary spastic paraplegia

**DOI:** 10.1186/s13023-022-02451-1

**Published:** 2022-07-29

**Authors:** Kathrin S. Utz, Zacharias Kohl, Dominique Cornelius Marterstock, Arnd Doerfler, Jürgen Winkler, Manuel Schmidt, Martin Regensburger

**Affiliations:** 1 Department of Neurology, Friedrich-Alexander-Universität Erlangen-Nürnberg (FAU), Erlangen, Germany; 2 Department of Molecular Neurology, FAU, Schwabachanlage 6, 91054 Erlangen, Germany; 3 Center for Rare Diseases (ZSEER), University Hospital Erlangen, Erlangen, Germany; 4 Department of Neuroradiology, FAU, Erlangen, Germany; 5 Present Address: Department of Neurology, University of Regensburg, Regensburg, Germany

**Keywords:** SPG11, Verbal fluency, Brain morphometry, Spastic paraplegia, Neuropsychology

## Abstract

**Background:**

*SPG11*-linked hereditary spastic paraplegia is characterized by multisystem neurodegeneration leading to a complex clinical and yet incurable phenotype of progressive spasticity and weakness. Severe cognitive symptoms are present in the majority of SPG11 patients, but a systematic and multidimensional analysis of the neuropsychological phenotype in a larger cohort is lacking. While thinning of the corpus callosum is a well-known structural hallmark observed in SPG11 patients, the neuroanatomical pattern of cortical degeneration is less understood. We here aimed to integrate neuropsychological and brain morphometric measures in SPG11.

**Methods:**

We examined the neuropsychological profile in 16 SPG11 patients using a defined neuropsychological testing battery. Long-term follow up testing was performed in 7 patients. Cortical and subcortical degeneration was analyzed using an approved, artificial intelligence based magnetic resonance imaging brain morphometry, comparing patients to established reference values and to matched controls.

**Results:**

In SPG11 patients, verbal fluency and memory as well as frontal-executive functions were severely impaired. Later disease stages were associated with a global pattern of impairments. Interestingly, reaction times correlated significantly with disease progression. Brain morphometry showed a significant reduction of cortical and subcortical parenchymal volume following a rostro-caudal gradient in SPG11. Whereas performance in memory tasks correlated with white matter damage, verbal fluency measures showed strong associations with frontal and parietal cortical volumes.

**Conclusions:**

The present data will help define neuropsychological and imaging read out parameters in early as well as in advanced clinical stages for future interventional trials in SPG11.

**Supplementary Information:**

The online version contains supplementary material available at 10.1186/s13023-022-02451-1.

## Background

Hereditary spastic paraplegias (HSP) are a heterogeneous group of rare genetic diseases characterized by progressive spasticity and weakness of the legs [1]. Biallelic pathogenic variants in *SPG11* cause the most frequent form of autosomal-recessive HSP leading to a multisystem neuronal degeneration [2–4]. While there is a substantial phenotypic variability, an early cognitive deficit is present in the majority of patients consequently followed by progressive motor symptoms in childhood or adolescence [5]. As there is no causal treatment present at time, SPG11 HSP results in early loss of ambulation, caregiver dependence, and premature death in mid-adulthood [6, 7].

While the common motor phenotype of HSPs is well validated applying the Spastic Paraplegia Rating Scale (SPRS [8]) as primary motor outcome measure in clinical studies of HSP, there are no standardized measures of neuropsychological symptoms linked to cognitive decline and progressive brain atrophy in SPG11. In particular, cognitive impairment has long been recognized as an important symptom in SPG11 [9, 10]. Most small case series, however, employed measures of intelligence like the Wechsler Intelligence Scale (WAIS) or global cognitive tests like the Mini-Mental State Examination (MMSE [11]), Montral Cognitive Assessment (MoCA [12]) or Addenbrooke’s Cogntitive Examination (ACE [13]). However, a more detailed neuropsychological evaluation in a larger patient cohort is missing in order to comprehensively characterize the complex and multidimensional phenotype of this devastating motor neuron disease.

Likewise, the known imaging hallmarks of SPG11 are a thin corpus callosum and periventricular white matter hyperintensities [2, 3, 10, 14], but a comprehensive depiction of the atrophy pattern and its relation to the clinical and neuropsychological phenotype is scarce. Furthermore, previous MRI studies of SPG11 patients used VBM or ROI approaches being limited to provide an unbiased volumetric approach to measure distinct grey and white matter regions. Of note, a recent case series in SPG11 described a predominant subcortical degeneration using different kinds of ROI based analyses [15].

The aim of this study was to comprehensively characterize the cognitive and cerebral atrophy phenotype in a well-defined cohort of SPG11 patients in order to (1) describe the neuropsychological profile of SPG11, (2) depict the progressive impairment in specific cognitive tasks, (3) quantify atrophy of different predefined brain regions, and (4) ultimately correlate the neuropsychological with the cerebral phenotype. The determination of a distinctive pattern of cortical and subcortical degeneration in SPG11 aids the diagnosis and is essential for a specific definition of potential clinical stages in regard to disease progression and for defining important outcome measures for future interventional studies in SPG11.

## Methods

### SPG11 cohort, clinical characteristics and neuropsychological assessment

Patients were recruited during regular follow-up visits within the HSP outpatient center of the Center for Rare Movement Disorders at the Department of Molecular Neurology, University Hospital Erlangen between 2016 and 2020. The study was approved by the local ethics committee (No. 259_17B and 347_17B), and all participants and legal guardians (if applicable) provided a written informed consent. Inclusion criteria were a clinically and genetically confirmed diagnosis of *SPG11*-linked HSP and the ability to participate in a defined set of cognitive tests. Motor examination included rating by the Spastic Paraplegia Rating Scale (SPRS [8]) that ranges from 0 to 52 and increases with disease severity. The Montreal Cognitive Assessment (MoCA [12]) was performed on 14 of the 16 patients to screen the overall cognitive status. The maximum score is 30 with patients scoring below 26 being considered as cognitively impaired.

Detailed demographic and clinical characteristics of the study cohort are summarized in Table 1. Details for neuropsychological assessments are given in the Additional file 1: supplemental Methods.

**Table 1:** Clinical and genetic characteristics of SPG11 patients

Patient no.	Gender	Age at onset (years)	Disease duration (years)	SPRS	MoCA	*SPG11* variants	Imaging results	Additional symptoms
1	m	15	7	21	5	c.733_734del (exon 4, p.M245fs)/c.4306_4307del (exon 25, p.Q1436fs)	TCC, enlarged ventricles	Bulbar speech, parkinsonism
2	f	1	22	21	17	c.3075dupA (exon 17, p.E1026fs)/c.6204A>G (exon 32, p.V2057Dfs)	TCC, periventricular white matter changes	Bulbar speech, epilepsy
3 (twin of 2)	f	1	22	17	15	c.3075dupA (exon 17, p.E1026fs)/c.6204A>G (exon 32, p.V2057fs)	TCC, periventricular white matter changes	Bulbar speech, neuropathy
4	f	19	29	36	14	c.267G>A (exon 2, p.W89*)/c.1457-2A>G (intron 6, splice acceptor)	TCC, frontal cortical atrophy, periventricular white matter changes	Bulbar speech, dysphagia, neuropathy
5	m	30	5	40	4	c.5623C>T (exon 30, p.Q1875*)homozygous	TCC, global atrophy, periventricular white matter changes	Bulbar speech, dysphagia, neuropathy
6	f	2	16	24	21	c.704_705del (exon 1, p.H235fs)/c.6832_6833del (exon 37, p.S2165fs)	TCC, frontoparietal atrophy, periventricular white matter changes	Bulbar speech, neuropathy, parkinsonism
7	f	20	20	37	–	c.3036C>A (exon 16, p.Y1012*)/c.5798_5798del (exon 30, p.A1933fs)	TCC, periventricular white matter changes	Bulbar speech, neuropathy
8 (sister of 7)	f	24	22	44	–	c.3036C>A (exon 16, p.Y1012*)/c.5798_5798del (exon 30, p.A1933fs)	TCC, periventricular white matter changes, frontal atrophy	Bulbar speech, neuropathy, edema, dysphagia
9	f	19	8	17	25	c.5623C>T (exon 30, p.Q1875*) homozygous	normal	Neuropathy
10	m	2	14	20	14	c.1203_1203delA (exon 6, p.D402fs) homozygous	TCC	Severe contractures, bulbar speech
11	m	42	3	10	28	c.255G>A (exon 1, p.W85*)/c.5381T>C (exon 30, p.L1794P)	normal	Neuropathy
12	m	9	10	27	10	c.2990T>A (exon 16, p.L997*)/c.4877_4878del (exon 28, p.F1626C)	TCC, periventricular white matter changes, frontal atrophy	Bulbar speech, parkinsonism
13	m	3	23	16	16	c.1951C>T homozygous (exon 10, p.R651*)	not available	None
14	m	3	22	31	8	c.190dupC (exon 1, p.Q64E)/c.704_705del (exon 4, p.H235fs)	TCC, periventricular white matter changes, frontal atrophy	Bulbar speech
15	f	15	6	14	30	c.2612dupG (exon 14, p.S871fs)/c.4434G>T (exon 25, p.? suspected splice variant)	TCC, periventricular white matter changes	None
16	f	1	22	16	19	c.2247del (exon 12, p.F750fs)/c.5623C>T (exon 30, p.Q1875*)	TCC, periventricular white matter changes, frontal atrophy	Neuropathy, bulbar speech, edema

*m* male, *f* female, *SPRS* spastic paraplegia rating scale, *MoCA* Montral Cognitive Assessment, *TCC* thinning of the corpus callosum

### Brain morphometry

High resolution MRI of the brain was performed in 12 patients using a 3.0 Tesla scanner (Magnetom Tim Trio, Siemens Healthineers, Erlangen, Germany) with a gradient field strength up to 45 mT/m (72 mT/m effective). Using the FDA-approved and CE-marked AI-Rad Companion Brain MR for Morphometry Analysis package (Siemens Healthineers), MPRAGE images underwent volumetric analysis. Segmentation quality was > 0.7 for all scans. To describe the pattern of brain atrophy in SPG11, the implemented algorithm was used first. It compares normalized morphometric values to reference values derived from a control group of the Alzheimer’s Disease Neuroimaging Initiative (ADNI [16]; http://adni.loni.usc.edu). This control group consists of 303 healthy controls (mean age 66 ± 19 years, age range 19–90 years, 51% of healthy individuals male) who underwent 3-Tesla cerebral MRI using the standardized ADNI protocol [17]. Normative ranges were calibrated on this population using a log-linear regression model taking into account the confounding effects of age and sex as covariates. Deviations from normative ranges of individual volume estimates were assessed by z-scores [18]. As clinically approved, relative values exceeding the 10th or 90th percentile were considered pathological. In order to analyze the brain atrophy pattern in a second approach, absolute morphometric volumes (in ml) were compared to a 1:1 age- and gender matched cohort of in-house controls (characteristics shown in Additional file 1: Table S1). For correlation to neuropsychological and clinical parameters, absolute values were normalized to intracranial volume.

### Statistics and data availability

As most data were not normally distributed and due to small sample sizes, non-parametric tests were used. Correlation analyses were calculated by Spearman’s rho (*r*). Raw values of baseline and follow-up testing were compared using the Wilcoxon signed-rank test. Absolute volumetric measures were compared using the unpaired Mann–Whitney test. *p* values < 0.05 were considered significant. Analyses were conducted in IBM SPSS 24 and graphs were generated in GraphPad Prism 8. Anonymized raw data are available from the corresponding authors upon reasonable request.

## Results

### Severe neuropsychological deficits are frequent in SPG11 patients

**Fig. 1:**
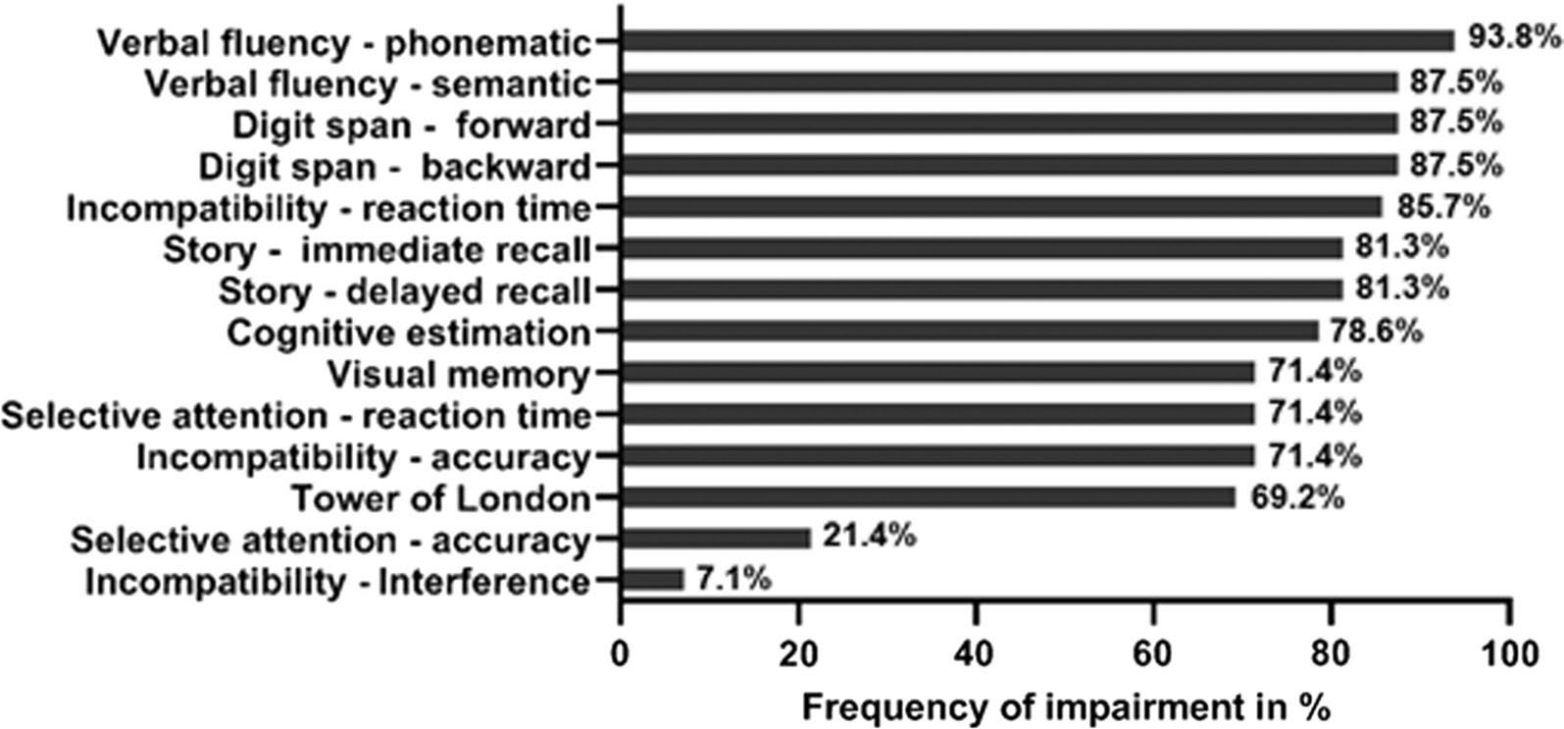
Frequency of impairment in different neuropsychological tests (n = 13 for Tower of London, n = 14 for visual memory, selective attention, incompatibility, cognitive estimation; n = 16 for all other tasks)

We recruited a total of 16 patients with a clinically and genetically confirmed diagnosis of *SPG11*-related HSP. All patients underwent a defined neuropsychological testing battery. Due to their advanced disease stage, data for visual memory, selective attention, incompatibility and cognitive estimation are missing in 2 patients, as well as for the Tower of London task in 3 patients, respectively. Figure 1 depicts the frequency of impairment for different neuropsychological tests, as defined by a result below a percentile rank of 16 (i.e. z < − 1) according to the respective test norms. The majority of SPG11 patients showed an impairment in more than 70% of the applied tests. Phonematic verbal fluency was most frequently impaired, followed by semantic verbal fluency, digit span forward, and digit span backward. Upon the global MoCA screening test, 12 out of 14 tested patients were impaired (86%), i.e., scoring below 26.

### Cognitive test performance correlates with motor deficits in SPG11

To examine whether patients at more advanced disease stages (i.e. with more severe motor impairment) exhibit more pronounced neuropsychological deficits, a sum score of tests scoring below a percentile rank of 16 was calculated for each patient. On average, patients were impaired in 9 out of 14 tests (n = 13, *M* = 9.77, *SD* = 3.37). There was a strong significant correlation between this sum score and the total SPRS score, indicating that a more severe motor phenotype of spastic paraplegia was associated with a higher number of impaired cognitive domains (*r* = 0.58, *p* = 0.038). Likewise, the global cognitive MoCA screening correlated significantly with SPRS score (*r* = 0.66, *p* = 0.01).

To identify those tests that correlated best with motor disease stage, raw scores of individual tests were used. There were strong significant correlations between total SPRS scores and story—immediate recall (*r* = − 0.63, *p* = 0.009), verbal fluency—semantic (*r* = − 0.77, *p* = 0.001), selective attention—reaction time (*r* = 0.73, *p* = 0.003), incompatibility—reaction time (*r* = 0.77, *p* = 0.001), and Tower of London (*r* = − 0.60, *p* = 0.039). These data imply that a more severe spastic paraplegia was associated with a worse performance in these neuropsychological tasks. The subscore of language domains in the MoCA test (sum of naming and repetition tasks, ranging from 0 to 5) correlated significantly with SPRS score (*r* = − 0.75, *p* = 0.002). There was no significant correlation between total SPRS scores and all other cognitive parameters (*p* > 0.05).

To identify those cognitive domains that are already affected at an early stage of motor impairment, patients were split based on the median SPRS score (25 points). Three patients with missing values were excluded, thus the sample size was 13. Five out of 6 patients scoring below the median showed impairment in the tasks digit span forward, digit span backward, and verbal fluency (both phonematic and semantic). These neuropsychological results suggest that verbal fluency and verbal short-term memory are linked to an early disease stage.

### Longitudinal course of motor and neuropsychological deficits

To characterize the progressive nature of SPG11, the testing battery was repeated after a mean interval of 24 months (range 12–44, n = 7). The median SPRS score of this sub-sample was 24 (IQR = 12) at the first examination and increased significantly to 29 (IQR = 17) at re-examination (z = − 2.37, *p* = 0.018). The global cognitive performance measured by MoCA however did not change significantly during this time period (z = − 0.83, *p* = 0.409, median = 16; IQR = 7 for both time points).

Concerning the course of neuropsychological impairment, the median sum score of tests scoring below a percentile rank of 16 was not significantly different between both examination time points (z = 0, *p* = 1).

When comparing performance in single tests, specifically reaction time in the selective attention task differed significantly between both time points (see Additional file 1: Table S2). Over time, reaction times increased by 50%, indicating a clinically relevant decrease in response speed. There was no significant correlation between reaction times and SPRS at both timepoints (baseline: *r* = 0.20, *p* = 0.670; follow-up: *r* = 0.27, *p* = 0.551) and also no correlation of the absolute changes in reaction times and SPRS values (*r* = 0.32, *p* = 0.498), indicating that the increase of reaction times may be independent from the worsened motor phenotype (Additional file 1: Fig. S1).

To sum up, while motor impairment worsened over time, there was no obvious change of overall cognitive impairment. However, we were able to detect a specific increase in reaction time in the computer-based selective attention task over time.

### Rostro-caudal pattern of cortical and subcortical atrophy in SPG11

**Fig. 2:**
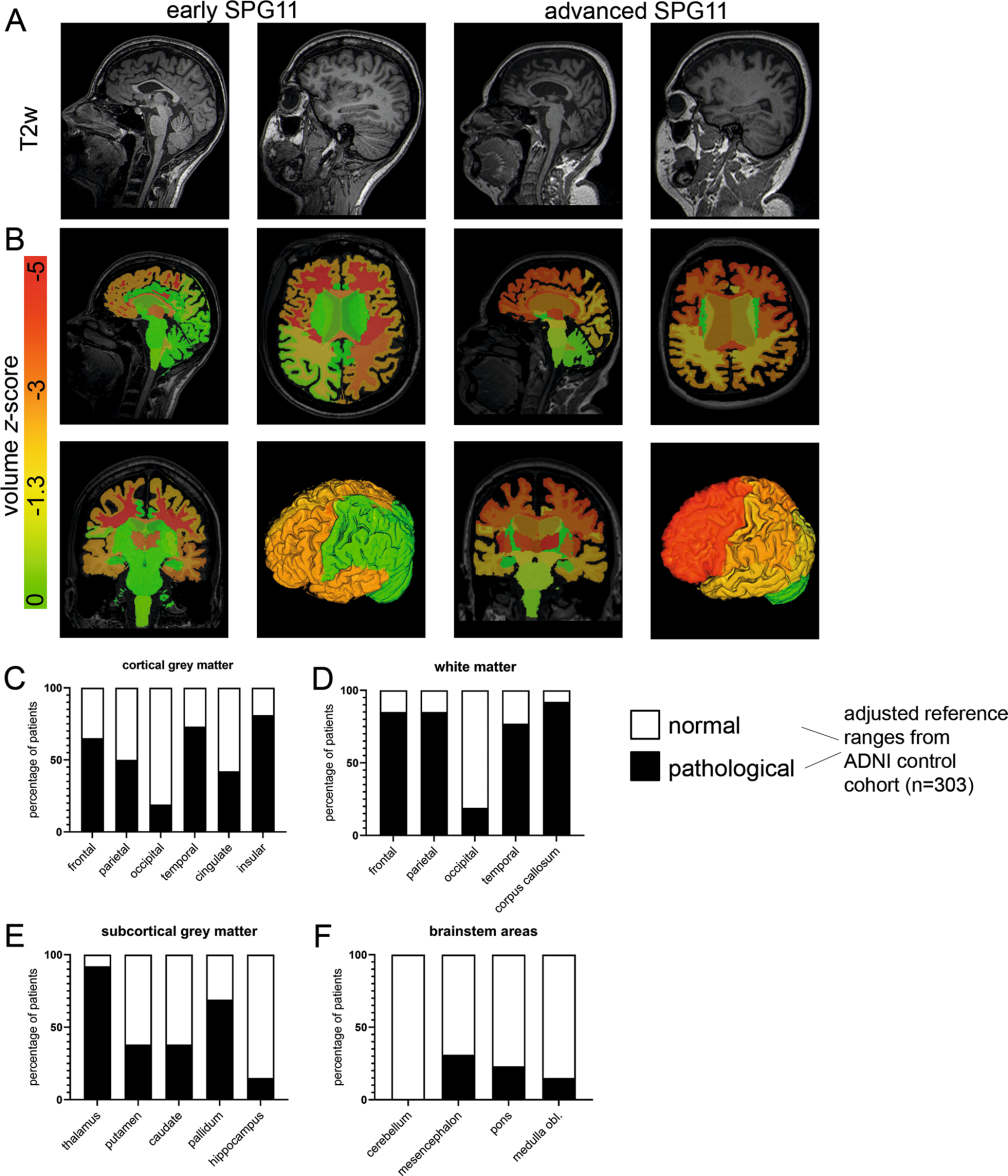
Brain volumetry in SPG11 patients relative to ADNI reference values. **A** Classical MRI signs of thin corpus callosum and cortical atrophy shown in an early, mildly affected SPG11 patient (individual #16 in Table 1; SPRS 16) and a severely affected SPG11 patient at an advanced stage of the disease (individual #4; SPRS 36). **B** Representative graphical depiction of volume z-scores of different cortical and subcortical regions of both patients. **C**–**F** Bar diagrams for different brain regions representing the proportional presence of a distinct structural phenotype, i.e. < 10% of age- and gender-matched control values derived from the ADNI control cohort

Among our cohort, 13 SPG11 patients underwent cerebral MRI at a 3.0-Tesla scanner. Most patients exhibited the prototypical signs of SPG11, i.e. thinning of the corpus callosum and cortical atrophy (Fig. 2A). The degeneration pattern of different grey and white matter areas was analyzed by a CE-marked volumetry tool recently approved for clinical use by the U.S. Food and Drug Administration. It was applied on the full brain MP-RAGE datasets. Using the implemented diagnostic algorithm, we first compared volumetric data to age- and gender-specific reference values established from the normative reference database for brain morphometry from the Alzheimer's Disease Neuroimaging Initiative (ADNI). As shown in the graphical representation of an early-stage patient and an advanced-stage patient (Fig. 2B), there was an inverse rostro-caudal gradient of brain atrophy involving both grey and white matter areas. Whereas the more rostral cerebral cortical areas showed a reduced volume in the majority of patients, parietal, cingulate and occipital cortical volumes were frequently within normal limits (Fig. 2C). Likewise, volumes of the corpus callosum and rostral white matter areas were reduced in most patients while occipital white matter volume was regular in 85% of patients (Fig. 2D). Interestingly, also subcortical grey matter areas were reduced in volume in a substantial proportion of patients, affecting the thalamus in 12/13 patients while sparing the hippocampus (Fig. 2E). On the other hand, cerebellar and brainstem volumes were decreased in few patients only (Fig. 2F).

**Fig. 3:**
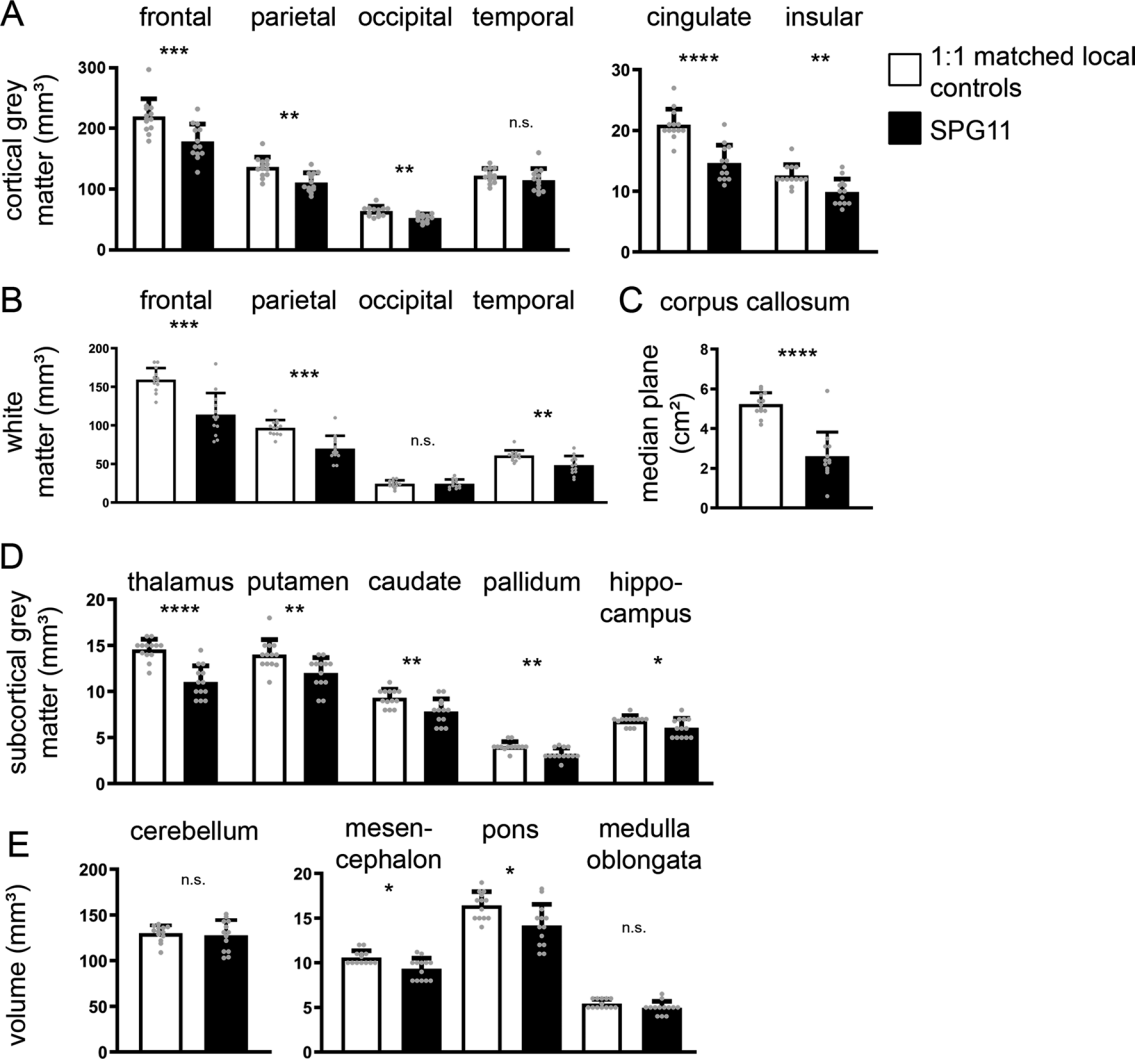
Brain volumetry in SPG11 compared to 1:1 age- and gender-matched in-house controls (n = 13, demographic characteristics shown in Additional file 1: Table S1) for different cortical and subcortical regions: (**A**) cortical regions, (**B**) white matter regions, (**C**) corpus callosum, (**D**) subcortical grey matter regions, (**E**) infratentorial regions. Bars represent means ± SD of absolute volume values in controls (white) and SPG11 patients (black). Means were compared by unpaired Mann–Whitney test. *p < .05; **p < .01; ***p < .001; ****p < .0001; *n.s.* not significant

In order to cross validate these data to a locally recruited control cohort, 13 age- and gender-matched control subjects underwent brain imaging at the identical MRI scanner. Comparing absolute volumes of different cortical and subcortical areas of SPG11 patients and these controls, we confirmed the severe decrease in cortical grey matter areas in SPG11 which was more pronounced in the frontal and parietal lobe (Fig. 3A). Concerning white matter areas, the reduction of white matter volume was also most pronounced within the frontal and parietal lobe (Fig. 3B). Analysis of the corpus callosum confirmed the structural hallmark of thinning of the corpus callosum in SPG11 (Fig. 3C). In line with the reference value analysis (Fig. 2F), also subcortical grey matter areas were significantly smaller when compared to our in-house controls, i.e. thalamus, putamen, caudate and pallidum (Fig. 3D). While cerebellar and medulla oblongata volumes were unchanged in SPG11, there was a small, but significant reduction of midbrain and pontine volume in SPG11 (Fig. 3E).

In summary, our morphometric analysis shows a specific pattern of brain degeneration in SPG11 with rostral (i.e., frontal, parietal and temporal) areas being predominantly affected, involving cortical and subcortical regions of both forebrain hemispheres.

### Cortical atrophy associates with cognitive rather than motor impairment

**Fig. 4:**
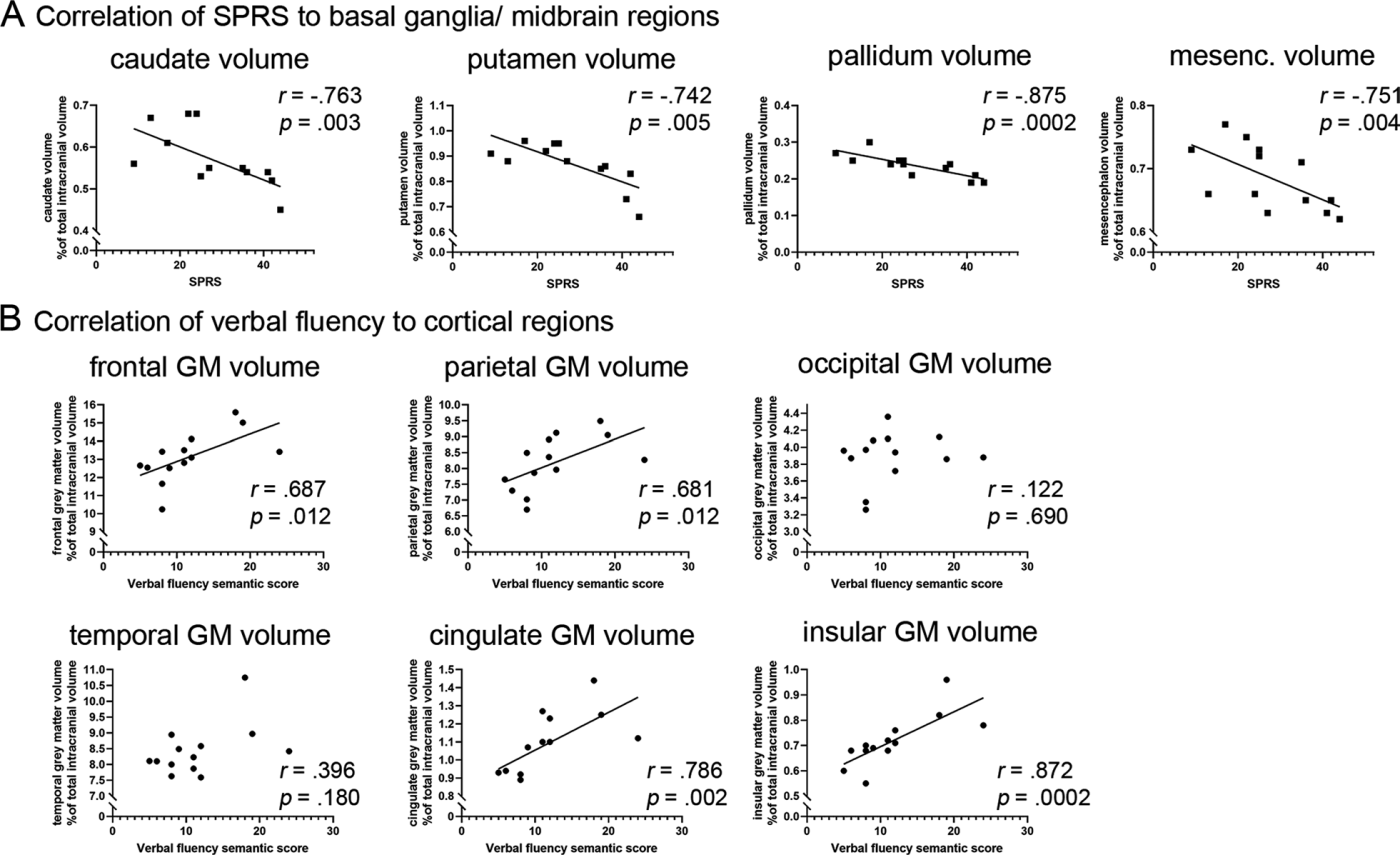
Correlation of grey matter atrophy to motor and cognitive function in SPG11. **A** Motor function (as measured by the SPRS score) correlates to basal ganglia and mesencephalic atrophy. **B** Verbal fluency (as measured by the verbal fluency semantic score) correlates to fronto-parietal cortical atrophy. *r* Spearman’s rho correlation coefficient, *SPRS* spastic paraplegia rating scale, *GM* grey matter

We next asked whether the degree of atrophy of specific brain regions correlates with motor functions or specific neuropsychological outcomes. As the SPRS score mostly reflects motor function, we first correlated imaging data to total SPRS scores. Interestingly, frontal and parietal grey matter and white matter regions showed no significant correlation to SPRS scores (complete set of correlations shown in Additional file 1: Table S3). There were strong inverse correlations of SPRS scores to subcortical motor region volumes (Fig. 4A), i.e. to putamen (*r* = − 0.742, *p* = 0.005), caudate (*r* = − 0.763, *p* = 0.003) and pallidum (*r* = − 0.873, *p* = 0.0002) and to the mesencephalon (*r* = − 0.751, *p* = 0.004).

Importantly, correlation analyses identified no specific brain regions that may account for neuropsychological deficits in SPG11 in general (Additional file 1: Table S3). Rather, distinct neuropsychological measures correlated with volumes of specific brain regions. For example, performance in the verbal memory test (‘Story Immediate’ and ‘Story Delayed’) correlated with parietal and temporal white matter volumes (Additional file 1: Table S3), both involved in memory processes. As an additional example, the ‘verbal fluency semantic’ score correlated significantly with those cortical grey matter areas that were also most affected on volumetry, i.e. frontal, parietal, cingulate and insular cortex (Fig. 4B).

## Discussion

The aim of this study was to provide a detailed neuropsychological and MRI volumetric characterization of the progressive forebrain degeneration in *SPG11*-linked HSP. A detailed understanding of the natural history of this devastating disorder is an important prerequisite for the identification of robust outcome parameters in order to design interventional trials, a challenging task in rare diseases. Clearly, disease mechanisms and the phenotype of SPG11 HSP are different from pure HSPs [19]. This highlights the importance of genotype-specific studies in HSP beyond motor correlations.

### Neuropsychological profile in SPG11

An impaired cognitive performance was present to different degrees in all patients. Specifically, verbal fluency, verbal memory, and executive functions were frequently impaired, reflecting a profound frontotemporal deficit. Overall, neuropsychological impairment correlated with the severity of the motor phenotype. Particularly, reaction times showed a significant worsening on follow-up testing and may thus constitute a robust digital biomarker in natural history studies and interventional studies. Verbal fluency and verbal short-term memory affected early in the disease course may indicate the onset of the neuropsychological deficit or alternatively reflect a specific neurodevelopmental phenotype.

Impaired scores on the global scales such as MMSE and WAIS have been previously reported in SPG11, as comprehensively summarized in Additional file 1: Table S4. Extending these findings of an overall cognitive impairment in SPG11 patients, we here describe a phenotype of impaired verbal fluency and verbal memory in > 80% of SPG11 patients indicating a frontotemporal neuropsychological phenotype. Severe cognitive impairment was also described in other less frequent genetic types of complicated HSP including SPG15 and the AP4-related HSP genes [5, 20, 21], but seems to be absent or mild in most patients with pure HSP, including SPG4, SPG5, and SPG7 [22].

Concerning global cognitive performance as assessed by MoCA, the large majority of SPG11 patients (12/14, i.e. 86%) were impaired, which is in line with Faber et al. [15] reporting 84% of patients classified as demented according to the global ACE-R score. A single SPG11 patient (#11, Table 1) exhibited neuropsychological impairment in distinct subtests only, which matches the previously reported mild phenotype associated with his specific missense variant [23]. No additional genotype phenotype correlations were observed as the majority of patients carried biallelic loss of function variants.

### Artificial intelligence based volumetry patterns of cortical atrophy in SPG11

For morphometric analyses, we applied the “AI-Rad Companion Brain MR for Morphometry Analysis” tool, which determines the outlines of different lobes and the separation between white and grey matter areas using artificial intelligence based algorithms. In contrast to the classical methods of voxel based morphometry (VBM) and region of interest (ROI) based volumetry, this method provides unbiased volumetric measures of different brain areas. Although volumetry analyses may be less powerful to demonstrate grey matter alterations [24], our data clearly show a grey matter phenotype in SPG11. The implemented reference values (normalized for intracranial volume) are derived from a control cohort within ADNI and importantly, its application has been approved for regular clinical use. Using a cohort of own local, gender and age matched controls, we cross validate the frontotemporal predominant grey and white matter atrophy in SPG11 and thus also provide support for the validity of the ADNI cohort as controls for volumetric studies in HSP.

Previous imaging studies on SPG11 predominantly described involvement of white matter regions and the basal ganglia. Our observation of a rostro-caudal gradient of white matter volume decrease in SPG11 matches findings of previous studies: a frontal predominant decrease of fractional anisotropy was described in a study on 5 SPG11 patients [25]. Widespread and severe white matter damage was also observed in a combined VBM/DTI study on 24 SPG11 patients, with inverse correlations of ACE-R scores and fractional anisotropy for most tracts [15, 26]. In this VBM-based analysis, cortical grey matter involvement was limited to motor regions and associative cortices whereas subcortical grey matter regions were predominantly affected. Likewise, in a study consisting of two mildly demented and three severely demented SPG11 patients, grey matter atrophy was mainly restricted to the basal ganglia and involved small areas within the precentral and postcentral gyrus only [27]. The different approach for cortical volumetry providing a higher sensitivity of artificial intelligence based lobe-wise morphometry may explain the detected widespread pattern of cortical atrophy. In fact, profound cortical degeneration was reported in a total of 3 post-mortem cases of SPG11 HSP [6, 28]. Both studies describe a severe degeneration predominantly affecting layer 5 neurons in the frontal lobe confirmed by our MRI analyses.

**Fig. 5:**
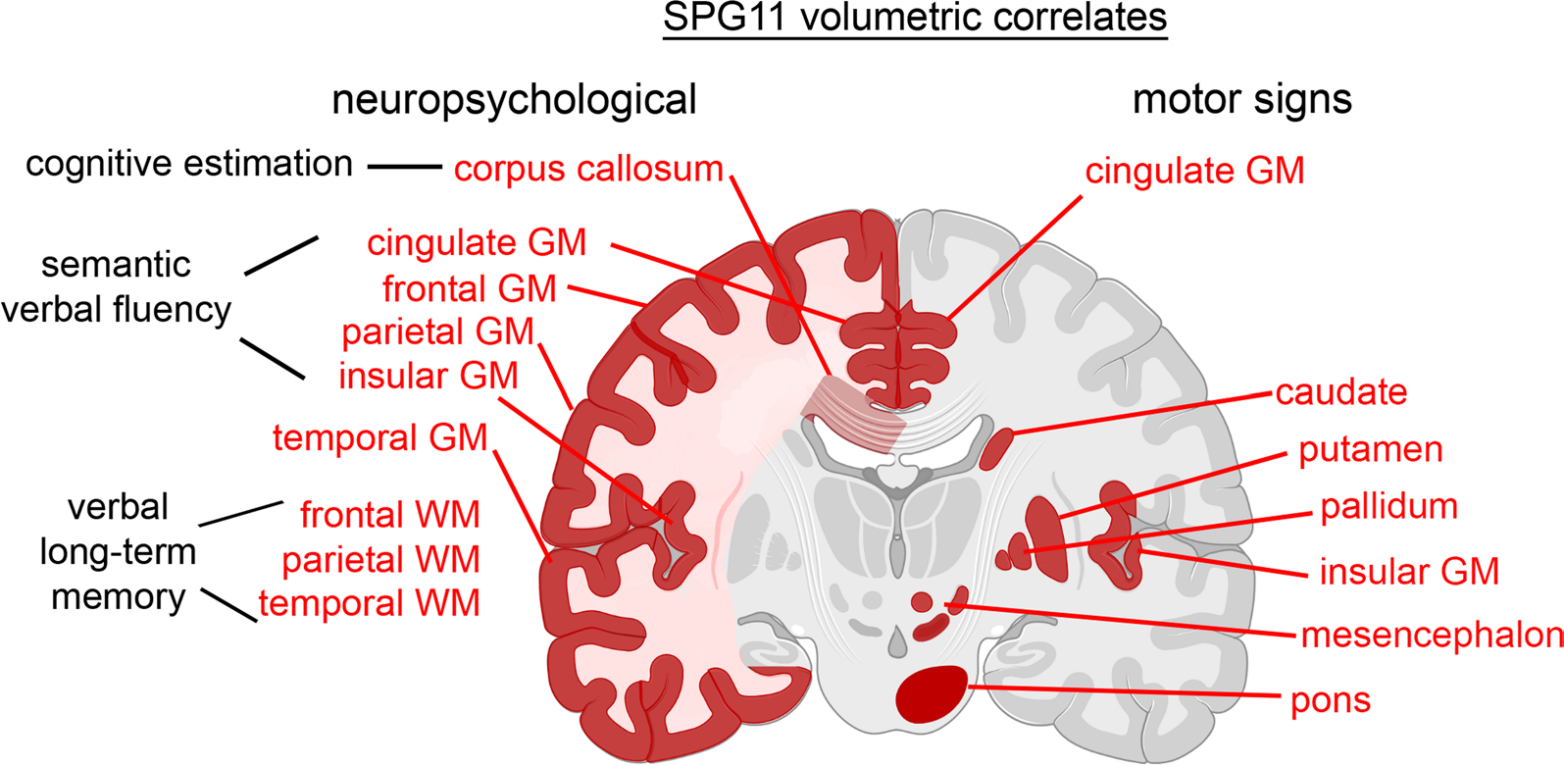
Volumetric loss in SPG11 (highlighted in red), separated by the correlation pattern to neuropsychological parameters (left) and SPRS (right)

The observed atrophy pattern of the frontal and temporal cortex correlates to the neuropsychological deficits of verbal fluency, verbal memory and executive functions. It is also in line with the frontotemporal cognitive deficit reported in the literature [29, 30]. We additionally identified an atrophy of the parietal cortex, an area that is involved in memory processes as well as visuospatial abilities. The correlation analysis of single cognitive test parameters to cortical volume parameters are partly as expected from lobe-specific functions; i.e. semantic verbal fluency correlated with frontal areas [31, 32] and verbal long-term memory with temporal areas [33, 34]. A schematic overview of volumetric measures and neuropsychological and motor impairment is summarized in Fig. 5. Due to our limited sample size, these results should be cautiously interpreted and be validated in future multicenter studies. It is also important to note the heterogeneity of the analyzed cohort with regards to age and disease duration. In fact, retrospective determination of age at onset from the patients’ history is often equivocal: most families reported insidiously progressive cognitive impairment already during childhood which may have remained unrecognized in some families.

Our data add to previous evidence of dual neurodevelopmental and -degenerative disease mechanisms in SPG11 [4, 15]. Developmental deficits may be linked to an early thinning of the corpus callosum and partly be associated with those neuropsychological measures being affected during the entire disease course, i.e. verbal fluency and digit span tests. Clearly, progressive neurodegeneration causes motor impairment and grey matter atrophy which is supported by our correlations of SPRS and subcortical grey matter volumetry. The underlying mechanism of the increased susceptibility of the more rostral areas remains unknown. However, this pattern is characteristic of frontotemporal dementias. Of note, accumulation of insoluble cytoplasmic TDP-43 (the histopathological hallmark of amyotrophic lateral sclerosis) was also described in a post mortem case of SPG11 [35], and the clinical overlap of SPG11 with amyotrophic lateral sclerosis has been described before [7].

### Progression of disease-specific impairments

In SPG11, motor and cognitive functions deteriorate over time [2, 10, 36]. We also observed worsening of motor function in our cohort, as indicated by progression of the SPRS score in those patients that underwent follow up analysis. The observed decrease of reaction time in computer-based attention tasks may be caused by worsened motor function as other global cognitive functions did not change over time. However, the progression rates of SPRS and reaction time were not significantly correlated to each other, indicating that reaction time might constitute an independent, longitudinal measure of cognitive impairment. Moreover, cognitive functions were inversely associated with SPRS in the overall sample, suggesting that cognitive impairment is at least partly progressive in parallel to the motor deficit. The major impact of frontotemporal neuropsychological impairment on patients’ quality of life and social interaction underlines the relevance of the neuropsychological and related imaging parameters as meaningful outcome measures [37].

## Conclusions

We report a rostro-caudal pattern of cortical and subcortical atrophy in SPG11 accompanied with severe motor impairment and neuropsychological impairment, especially of verbal fluency, verbal memory and executive functions. Our data suggest that reaction times and MRI cortical volumetric measures are important outcome measures in future interventional trials for SPG11. As the presented artificial intelligence based volumetric analysis is feasible across imaging platforms, future functional and structural studies in SPG11 are possible in a multicentric and longitudinal manner in order to validate these findings and to reduce potential bias across academic centers.

## Supplementary Information

**Additional file 1. Figure S1:** Individual progression of both Reaction Time and SPRS in SPG11. **Table S1**: Demographic characteristics of SPG11 and in-house control imaging cohorts. **Table S2**: Longitudinal results of cognitive parameters (n = 7 patients, mean interval of 24 months). **Table S3**: Correlation coefficient (r) matrix of imaging data with neuropsychological and motor measures. **Table S4**: Overview of previous studies applying neuropsychological testing in SPG11 HSP. **Supplemental Methods. Supplemental References.**

## Data Availability

The datasets used and analysed during the current study are available from the corresponding author on reasonable request.
