# Supplementary material for: Neuropsychology and MRI correlates of neurodegeneration in SPG11 hereditary spastic paraplegia
Source: Orphanet J Rare Dis. 2022 Jul 29;17:301. doi: 10.1186/s13023-022-02451-1 (PMC9336101; doi:10.1186/s13023-022-02451-1)
Supplement: Supplementary file 1 — Additional file 1. Figure S1: Individual progression of both Reaction Time and SPRS in SPG11. Table S1: Demographic characteristics of SPG11 and in-house control imaging cohorts. Table S2: Longitudinal results of cognitive parameters (n = 7 patients, mean interval of 24 months). Table S3: Correlation coefficient (r) matrix of imaging data with neuropsychological and motor measures. Table S4: Overview of previous studies applying neuropsychological testing in SPG11 HSP. Supplemental Methods. Supplemental References. [file 13023_2022_2451_MOESM1_ESM.docx]

**Supplemental Figure 1. Individual progression of both Reaction Time and SPRS in SPG11.**


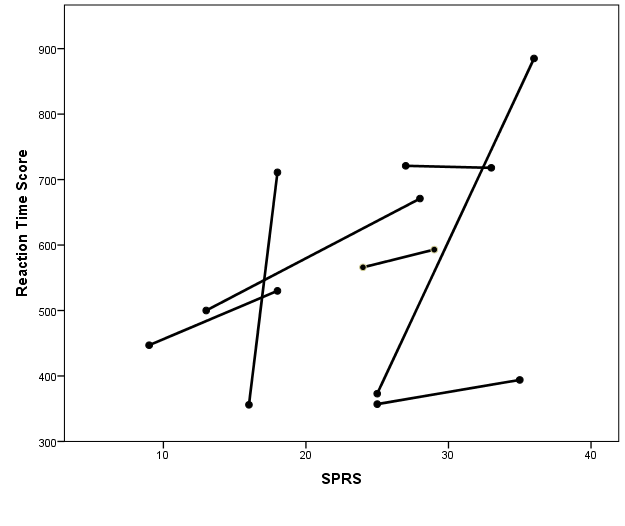


Illustration of the progression of SPRS and reaction time. Results from individual patients are connected by lines. While both parameters worsened over time in most patients, the degree of worsening did not correlate between both parameters.

**Supplemental Table 1:** Demographic characteristics of SPG11 and in-house control imaging cohorts

| **Parameter (mean ±SD)** | **SPG11 HSP**  **n=13** | **Controls**  **n=13** | **P value** |
| --- | --- | --- | --- |
| Age (y) | 27.6 ± 10.1 | 27.7 ± 10,2 | >0.05 |
| Gender (male : female) | 5:8 | 5:8 | >0.05* |
|  |  |  |  |

**Supplemental Table 2:** Longitudinal results of cognitive parameters (n = 7 patients, mean interval of 24 months).

| **Cognitive parameter** | **Examination 1**  **Median (IQR)** | **Examination 2**  **Median (IQR)** | **z** | ***p*** |
| --- | --- | --- | --- | --- |
| Digit span - forward | 4 (1) | 4 (1) | 0.0 | 1 |
| Digit span - backward | 3 (1) | 3 (1) | -1.89 | .59 |
| Story - immediate recall | 2.5 (7) | 2 (9,5) | -0.11 | .92 |
| Story - delayed recall | 1.5 (3.5) | 2 (9,5) | -1.53 | .13 |
| Visual memory | 6 (3) | 6 (2) | -1.71 | .09 |
| Verbal fluency - semantic | 12 (7) | 12 (8) | -0.85 | .40 |
| Verbal fluency - phonematic | 3 (4) | 4 (5) | -0.85 | .40 |
| Selective attention - reaction time | 447 (209) | 671 (188) | -2.20 | **.03** |
| Selective attention - accuracy | 2 (10) | 3 (5) | -1.67 | .24 |
| Incompatibility - reaction time | 640 (420) | 636 (286) | -1.18 | .24 |
| Incompatibility - accuracy | 11 (21) | 3 (22) | -1.87 | .06 |
| Incompatibility - interference | 1.97 (5.49) | 2.68 (6.96) | -0.17 | .87 |

Abbreviation: *IQR* interquartile range; p < 0.05 is highlighted in bold.

**Supplemental Table 3:** Correlation coefficient (*r*) matrix of imaging data with neuropsychological and motor measures.

|  | GM frontal | GM parietal | GM occipital | GM temporal | GM cingulate | GM insular | WM frontal | WM parietal | WM occipital | WM temporal | thalamus | putamen | caudate | pallidum | hippocampus | cerebellum | mesencephalon | pons | medulla obl. | corpus callosum |
| --- | --- | --- | --- | --- | --- | --- | --- | --- | --- | --- | --- | --- | --- | --- | --- | --- | --- | --- | --- | --- |
| SPRS | -0,377 | -0,424 | -0,171 | -0,063 | **-0,687*** | **-0,747**** | -0,135 | -0,388 | -0,193 | -0,539 | -0,428 | **-0,742**** | **-0,763**** | **-0,873***** | -0,265 | -0,476 | **-0,751**** | **-0,659*** | -0,506 | -0,270 |
| Digit span forward | -0,168 | -0,207 | -0,201 | -0,054 | -0,199 | -0,326 | 0,378 | 0,168 | -0,138 | -0,087 | -0,351 | **-0,582*** | **-0,590*** | **-0,603*** | -0,321 | **-0,768**** | **-0,622*** | -0,515 | -0,271 | -0,057 |
| Digit span backward | -0,026 | 0,092 | -0,011 | 0,060 | 0,115 | 0,063 | -0,160 | 0,275 | 0,298 | 0,120 | -0,225 | 0,180 | 0,222 | -0,112 | 0,113 | 0,229 | 0,153 | 0,257 | 0,038 | 0,301 |
| Story - immediate recall | 0,287 | 0,264 | 0,025 | 0,186 | **0,577*** | **0,738**** | 0,440 | **0,818**** | **0,634*** | **0,882***** | 0,441 | 0,256 | **0,709**** | 0,398 | 0,368 | 0,181 | 0,297 | 0,429 | 0,021 | 0,542 |
| Story - delayed recall | 0,070 | -0,017 | -0,267 | 0,331 | 0,134 | 0,389 | **0,645*** | **0,741**** | **0,676*** | **0,755**** | 0,303 | -0,178 | 0,451 | -0,065 | 0,010 | -0,121 | -0,178 | 0,041 | -0,208 | 0,491 |
| Visual memory | 0,028 | -0,023 | 0,097 | 0,120 | 0,536 | 0,560 | **0,647*** | **0,739*** | 0,365 | 0,601 | -0,206 | 0,313 | **0,704*** | 0,391 | -0,334 | 0,092 | 0,219 | 0,175 | -0,058 | 0,554 |
| Verbal fluency - semantic | **0,687*** | **0,681*** | 0,122 | 0,396 | **0,786**** | **0,872***** | 0,399 | 0,526 | 0,332 | 0,607 | 0,554 | **0,612*** | **0,588*** | **0,584*** | 0,076 | 0,233 | **0,577*** | 0,537 | 0,499 | 0,260 |
| Verbal fluency - phonematic | 0,168 | 0,115 | -0,121 | 0,182 | 0,331 | 0,497 | 0,421 | **0,651*** | 0,513 | 0,533 | 0,213 | 0,138 | 0,252 | 0,260 | 0,027 | 0,104 | 0,391 | 0,475 | 0,194 | **0,581*** |
| Selective attention - reaction time | -0,318 | -0,300 | -0,191 | 0,073 | -0,315 | -0,169 | -0,127 | 0,173 | 0,445 | 0,127 | -0,110 | **-0,662*** | 0,073 | -0,537 | 0,524 | -0,073 | -0,449 | -0,156 | **-0,613*** | 0,045 |
| Selective attention - accuracy | 0,246 | 0,367 | 0,343 | 0,034 | 0,150 | -0,152 | -0,285 | -0,488 | -0,555 | -0,565 | -0,349 | **0,625*** | -0,185 | 0,103 | -0,459 | 0,198 | 0,129 | -0,215 | 0,299 | -0,328 |
| Incompatibility - reaction time | -0,173 | -0,064 | -0,073 | 0,118 | **-0,749*** | **-0,674*** | **-0,691*** | **-0,700*** | -0,091 | **-0,773**** | 0,005 | -0,175 | -0,606 | -0,352 | -0,018 | 0,227 | -0,124 | -0,106 | 0,151 | -0,173 |
| Incompatibility - accuracy | -0,292 | -0,196 | 0,014 | **-0,708*** | 0,046 | -0,403 | -0,361 | -0,365 | **-0,694*** | -0,201 | -0,303 | 0,039 | -0,323 | -0,023 | 0,446 | -0,242 | -0,161 | -0,231 | -0,186 | -0,329 |
| Interference | -0,408 | -0,482 | -0,556 | -0,630 | -0,408 | -0,542 | -0,482 | -0,667 | -0,630 | -0,259 | -0,299 | -0,411 | -0,148 | -0,192 | 0,482 | -0,148 | -0,374 | -0,486 | -0,355 | -0,593 |
| Cognitive Estimation | -0,050 | -0,155 | -0,073 | 0,333 | 0,165 | 0,371 | 0,511 | 0,511 | **0,662*** | 0,470 | 0,132 | 0,058 | 0,403 | 0,288 | -0,194 | 0,237 | 0,140 | 0,113 | -0,189 | **0,772**** |
| Tower of London | -0,402 | -0,496 | -0,077 | -0,137 | 0,365 | 0,462 | 0,573 | **0,778*** | 0,410 | **0,693*** | -0,217 | 0,131 | **0,732*** | 0,485 | -0,109 | 0,231 | 0,293 | 0,401 | -0,203 | **0,769*** |

Bold *r* values indicate significant correlations (**p* < 0.05; ***p* < 0.01; ****p* < 0.001); grey color indicates significant *r* values > 0.7 or < -0.7 considered as strong correlations.

**Supplemental Table 4:** Overview of previous studies applying neuropsychological testing in SPG11 HSP

| **Reference** | **Number of SPG11 patients** | **Age of patients**  **in years** | **Disease duration**  **in years** | **Cognitive tests** | **Results** |
| --- | --- | --- | --- | --- | --- |
| deBot et al. 2013 [1] | 18 | Mean = 26 (range 13–46) | Mean = 7.9 | WAIS | 5 patients borderline MR (IQ = 70-79); 2 mild MR (IQ = 50/55-70); 6 borderline to mild MR; 2 moderate MR (IQ = 35-40-55), 2 not specified; 14 patients showed cognitive decline |
| Cao et al. 2013 [2] | 2 | Case 1 = 19  Case 2 = 23 | Case 1 = 5  Case 2 = 7 | MMSE; WAIS; ACE-R; AVLT; CFT; CWT; SDMT; CSDD; NPI; CFT; TMT-B; SCWT | Mild impairment of memory,  Executive functions and processing speed. |
| Crimella et al. 2009 [3] | 9 | Ages are generalised in ranges to keep anonymity = 19–64 | Age at onset: range = 5–50 | WAIS/WISC | 2 patients had normal IQ  5 patients: IQ = 45-65; reduced |
| Denora et al. 2009 [4] | 40 | Mean = 22 (range: 12–46) | Mean = 7.9 | IQ (test not specified); MMSE; WMS „in the vast majority of patients“ | 87% had mental impairment with objective evidence in 20 patients; IQ = 53-69. Few cases initially showed a low-normal IQ but scored poorly at subsequent testing |
| Faber et al. 2018 [5] | 25 | 29 (range:18–49) | Mean = 13 | ACE-R | 51/100 (range 10–88); |
| Garaci et al. 2014 [6] | 4 | Mean = 25 (range: 17–29) | Mean = 15 | MMSE | 2 patients: MR / cognitive detoriation;  2 patients: moderate MR |
| Giannoccaro et al. 2014 [7] | 2 | Case 1 = 47  Case 2 = 53 | Case 1 = 5  Case 2 = 4 | MMSE | Case 1: MMSE = 26/30  Case 2: no data |
| Kim et al. 2009 [8] | 2 | Case 1 = 25  Case 2 = 21 | Case 1 = 10  Case 2 = 6 | Case 1: MMSE; FAB  Case 2: MMSE; CWT; word fluency test; RVALT | Case 1: MMSE = 28/30; FAB: 15/18  Case 2: MMSE = 30/30; severely impaired executive function and working memory |
| Li et al. 2015 [9] | 4 | Mean = 23 (range: 18–24) | Median = 4 | MMSE; MoCA | MMSE = 28-30/30; normal |
| Ma et al. 2014 [10] | 1 | 14 | unable to speak any words until he was 4 years old | WISC | IQ = 53; reduced |
| Jacinto-Scudeiro et al. 2019 [11] | 5 | Mean = 36 (standard deviation: 16.8) | Mean = 20 | MMSE; MoCA; FAS; verbal fluency; RVALT | All patients were impaired in all tests |
| Pan et al. 2013 [12] | 5 | Mean = 30 (range: 25–35) | Mean = 19 | WAIS; MMSE | WAIS = IQ: 47-50; reduced  MMSE = 13-19/30; impaired |
| Patel et al. 2016 [13] | 1 | 19 | 3 | MMSE | MMSE = 26/30 |
| Pensato et al. 2014 [14] | 16 | Mean = 28 (range: 20–49) | Mean age at onset = 20 | Not specified | 9 patients presented moderate to severe cognitive decline (IQ range 55–72),  7 cases had an IQ score within the normal range, but presented  deficit in memory and calculation tests |
| Schneider-Gold et al. 2017 [15] | 2 (twins) | 22 | delayed motor milestones with first walking at the  age of 3 | Not specified | Case 1: IQ = 74  Case 2: IQ = 80  Dyscalculia, mild dyslexia, verbal and non-verbal short term memory deficits, autobiographical deficits, partial executive dysfunction |
| Siri et al. 2011 [16] | 2 | Case 1 = 15  Case 2 = 28 | Case 1 = 7  Case 2 = 19 | WAIS/WISC; Digit span; Corsi’s block tapping; Luria memory word test, TMT-B; Tower of London; WCST; Token Test; Peabody picture vocabulary test; BNT; verbal fluency; visual-motor integration test | Executive dysfunction: memory consolidation, attainment of skills, attention, visual perception and organization, slowed information processing; case 1 also deficits in learning and strategy planning |
| Stromillo et al. 2011 [17] | 10 | Mean = 29 (range: 18–46) | Mean = 16.2 | MMSE | MMSE = 18-25/30; impaired |
| Vanderver et al. 2012 [18] | 4 | Mean = 26 (range: 24–29) | Case 1 = 22  Case 2 and 3 = childhood onset  Case 4 = 8 | WAIS | IQ = 59-76; reduced |

Abbreviations: *ACE-R* Addenbrooke’s Cognitive Examination-Revised; *AVLT* auditory verbal learning test; *BNT* Boston Naming Test; *CSDD* Cornell scale for depression in dementia; *CFT*, ReyeOsterrieth complex figure test; *CWT* Stroop colour words test; *FAB* Frontal Assessment Battery; *MMSE* Mini-Mental State Examination; *MoCA* Montreal Cognitive Assessment; *MR* mental retardation; *NPI* neuropsychiatric inventory; *RAVLT* Rey’s Verbal Auditory Learning Test, *SDMT* Symbol-Digit Modalities Test; *TMT-B* Trail Making Test B; *WAIS* Wechsler intelligence scale for adult; *WCST* Wisconsin Card Sorting Test; *WISC* Wechsler intelligence scale for children; *WMS* Wechsler Memory Scale

**Supplemental Methods**

*Clinical characteristics of the SPG11 patient cohort*

Overall, the cohort reflected all stages of SPG11 HSP; it comprised 9 female and 7 male patients, all of Caucasian descent, with a mean age of 29 years (range 16–48), a mean disease duration of 16 years (range 3–29), and a mean SPRS score of 24 (range 10–44). Clinically, spastic paraparesis was present in all 16 patients. 2/16 patients were able to walk without a walking aid and 7/16 patients were wheelchair bound. The mean MoCA score was 16 (range 4–30).

*Neuropsychological assessment*

Common standardized neuropsychological tests assessing memory, attention, language and executive functions were conducted by a psychologist (KSU). The test battery included digit span forward and backward from the German edition [19] of the Wechsler Memory Scale Revised Edition (WMS-R; [20]) to assess verbal short-term memory. Verbal long-term memory was measured via story immediate and delayed from the German version [21] of the Rivermead Behavioural Memory Test [22]. Visual memory was tested applying the pattern recognition task of the Berlin Amnesia Test [23]. Verbal and semantic fluency were assessed using the subtests ‘Animals’ and ‘S-words’ of the Regensburg Word Fluency Test (Regensburger Wortflüssigkeitstest; [24]). Subtests from the computerized German test battery, ‘Testbatterie zur Aufmerksamkeitsprüfung’ [25] were conducted to assess selective attention (subtest ‘Go/Nogo’) and interference tendency (subtest ‘incompatibility’). The ‘Test zum kognitiven Schätzen’ [26] requires the estimation of everyday variables (i.e., height, weight) and involves semantic memory and executive functions. The German version of the Tower of London test [27] was applied to assess planning abilities attributed to executive functions.

**Supplemental references**

1. Bot ST de, Burggraaff RC, Herkert JC, Schelhaas HJ, Post B, Diekstra A, et al. Rapidly deteriorating course in Dutch hereditary spastic paraplegia type 11 patients. European journal of human genetics : EJHG. 2013;21:1312–5.

2. Cao L, Rong T-Y, Huang X-J, Fang R, Wu Z-Y, Tang H-D, et al. Novel SPG11 mutations in Chinese families with hereditary spastic paraplegia with thin corpus callosum. Parkinsonism & related disorders. 2013;19:367–70.

3. Crimella C, Arnoldi A, Crippa F, Mostacciuolo ML, Boaretto F, Sironi M, et al. Point mutations and a large intragenic deletion in SPG11 in complicated spastic paraplegia without thin corpus callosum. Journal of medical genetics. 2009;46:345–51.

4. Denora PS, Schlesinger D, Casali C, Kok F, Tessa A, Boukhris A, et al. Screening of ARHSP-TCC patients expands the spectrum of SPG11 mutations and includes a large scale gene deletion. Human mutation. 2009;30:E500-19.

5. Faber I, Martinez ARM, Rezende TJR de, Martins CR, Martins MP, Lourenco CM, et al. SPG11 mutations cause widespread white matter and basal ganglia abnormalities, but restricted cortical damage. NeuroImage Clinical. 2018;19:848–57.

6. Garaci F, Toschi N, Lanzafame S, Meschini A, Bertini E, Simonetti G, et al. Diffusion tensor imaging in SPG11- and SPG4-linked hereditary spastic paraplegia. The International journal of neuroscience. 2014;124:261–70.

7. Giannoccaro MP, Liguori R, Arnoldi A, Donadio V, Avoni P, Bassi MT. Atypical late-onset hereditary spastic paraplegia with thin corpus callosum due to novel compound heterozygous mutations in the SPG11 gene. Journal of neurology. 2014;261:1825–7.

8. Kim S-M, Lee J-S, Kim S, Kim H-J, Kim M-H, Lee K-M, et al. Novel compound heterozygous mutations of the SPG11 gene in Korean families with hereditary spastic paraplegia with thin corpus callosum. Journal of neurology. 2009;256:1714–8.

9. Li Y, Mao C, Shi C, Song B, Wu J, Qin J, et al. Exome sequencing reveals novel SPG11 mutation in hereditary spastic paraplegia with complicated phenotypes. Journal of clinical neuroscience : official journal of the Neurosurgical Society of Australasia. 2015;22:1150–4.

10. Ma J, Xiong L, Chang Y, Jing X, Huang W, Hu B, et al. Novel mutations c.[5121_5122insAG]+[6859C>T] of the SPG11 gene associated with cerebellum hypometabolism in a Chinese case of hereditary spastic paraplegia with thin corpus callosum. Parkinsonism & related disorders. 2014;20:256–9.

11. Jacinto-Scudeiro LA, Machado GD, Ayres A, Burguêz D, Polese-Bonato M, González-Salazar C, et al. Are Cognitive Changes in Hereditary Spastic Paraplegias Restricted to Complicated Forms? Front Neurol. 2019;10:508.

12. Pan M-K, Huang S-C, Lo Y-C, Yang C-C, Cheng T-W, Yang C-C, et al. Microstructural integrity of cerebral fiber tracts in hereditary spastic paraparesis with SPG11 mutation. AJNR American journal of neuroradiology. 2013;34:990-6-S1.

13. Patel S, Sethi PK, Anand I, Batra A, Gupta P. Hereditary spastic paraplegia with a thin corpus callosum due to SPG11 mutation. Neurology India. 2016;64:171–2.

14. Pensato V, Castellotti B, Gellera C, Pareyson D, Ciano C, Nanetti L, et al. Overlapping phenotypes in complex spastic paraplegias SPG11, SPG15, SPG35 and SPG48. Brain. 2014;137:1907–20.

15. Schneider-Gold C, Dekomien G, Regensburger M, Schneider R, Trampe N, Krogias C, et al. Monozygotic twins with a new compound heterozygous SPG11 mutation and different disease expression. J Neurol Sci. 2017;381:265–8.

16. Siri L, Battaglia FM, Tessa A, Rossi A, Rocco MD, Facchinetti S, et al. Cognitive profile in spastic paraplegia with thin corpus callosum and mutations in SPG11. Neuropediatrics. 2010;41:35–8.

17. Stromillo ML, Malandrini A, Dotti MT, Battaglini M, Borgogni F, Tessa A, et al. Structural and metabolic damage in brains of patients with SPG11-related spastic paraplegia as detected by quantitative MRI. Journal of neurology. 2011;258:2240–7.

18. Vanderver A, Tonduti D, Auerbach S, Schmidt JL, Parikh S, Gowans GC, et al. Neurotransmitter abnormalities and response to supplementation in SPG11. Molecular genetics and metabolism. 2012;107:229–33.

19. Härting C, Markowitsch HJ, Neufeld H, Calabrese P, Deisinger K, Kessler J. Wechsler Gedächtnistest - revidierte Fassung. Bern: Huber; 2000.

20. Wechsler D. WMS-R: Wechsler Memory Scale - Revised (Manual). San Antonio: The Psychological Corporation; 1987.

21. Beckers K, Behrends U, Canavan A. Der Rivermead Behavioural Memory Test. Bury St. Edmunds: Thames Valley Test Company; 1992.

22. Wilson BA, Clare L, Cockburn J, Baddeley A, Tate R, Watson P. The Rivermead Behavioural Memory Test-Extended Version. Suffolk: Thames Valley Test Company; 1987.

23. Metzler P, Voshage J, Rösler P. Berliner Amnesie-Test: BAT; zur Diagnostik von anterograden mnestischen Störungen. Göttingen: Hogrefe; 2010.

24. Aschenbrenner S, Tucha O, Lange KW. Regensburger Wortflüssigkeits-Test: RWT. Göttingen: Hogrefe; 2000.

25. Zimmermann P, Fimm B. Testbatterie zur Aufmerksamkeitsprüfung (Vol. 2.3). Herzogenrath: Psytest; 2012.

26. Brand M, Kalbe E, Kessler J. Test zum kognitiven Schätzen: TKS. Göttingen: Beltz Test; 2002.

27. Tucha O, Lange KW. Turm von London - Deutsche Version. Göttingen: Hogrefe; 2004.
